# State Earned Income Tax Credits and Firearm Suicides

**DOI:** 10.1001/jamanetworkopen.2025.1398

**Published:** 2025-03-21

**Authors:** Nicole Asa, Alice Ellyson, Ali Rowhani-Rahbar

**Affiliations:** 1 Department of Epidemiology, School of Public Health, University of Washington, Seattle; 2 Firearm Injury and Policy Research Program, School of Medicine, University of Washington, Seattle; 3 Department of Pediatrics and Firearm Injury and Policy Research Program, School of Medicine, University of Washington, Seattle

## Abstract

**Importance:**

Firearm suicides are an increasing public health crisis and disproportionately impact low-income communities. The earned income tax credit (EITC) is the largest poverty alleviation program for working families with low income in the US.

**Objective:**

To evaluate the association of presence and generosity of state EITC policies with state-level firearm suicide rates.

**Design, Setting, and Participants:**

This ecological cohort study analyzed EITC data and suicide rates from 46 US states and Washington, DC, from 1981 to 2019. Data analysis was conducted from June 2022 to August 2024.

**Exposure:**

The exposure was the presence and generosity of state refundable EITCs (ie, amount of a refundable tax credit that exceeds income tax liability is refunded to taxpayers). The EITC generosity was measured as a continuous exposure quantified by percentage of the federal EITC offered by a state each year.

**Main Outcomes and Measures:**

The outcome was state-level annual firearm suicide rates. The association of state EITC implementation with firearm suicide rates was estimated using heterogeneity-robust 2-stage difference-in-difference (DID). All regression models used ordinary least squares, included state and year fixed effects with standard errors clustered at the state level, and adjusted for time-varying state-level covariates.

**Results:**

Individual-level data were not available in this study of state-level firearm suicide rates. During the study period, 20 states implemented a refundable EITC (generosity range, 3.4%-85%). In 2019, the final year of the study, the nationwide rate of firearm suicide was 7.29 per 100 000 persons. A statistically significant negative association was found between refundable EITCs and firearm suicide rates. Every 10% increase in refundable EITC generosity was associated with a decrease in the mean firearm suicide rate by 0.28 (95% CI, −0.48 to −0.08) cases per 100 000 person-years (relative decline, 4.3%; 95% CI, 1.5% to 7.0%). The mean firearm suicide rate was 0.49 (95% CI, −0.91 to −0.07) cases per 100 000 person-years fewer comparing postimplementation and preimplementation years among states that implemented a refundable EITC contrasting with the change during that time in states without a refundable EITC (relative decline, 7.9%; 95% CI, 2.7% to 12.9%).

**Conclusion:**

In this cohort study, the presence and generosity of state refundable EITCs were associated with a decrease in firearm suicide rates, supporting the growing body of literature highlighting the importance of antipoverty policies for reducing firearm suicide.

## Key Points

**Question:**

Are state earned income tax credits associated with firearm suicide rates?

**Findings:**

In this cohort study of 46 states and Washington, DC, every 10% increase in refundable earned income tax credit generosity was associated with 0.28 fewer cases of firearm suicide per 100 000 people per year, corresponding to a relative decline of 4.3%.

**Meaning:**

These findings suggest that implementation of more generous state earned income tax credit programs may be associated with reductions in firearm suicide rates.

## Teaser

This cohort study examines state earned income tax credit generosity and state-level firearm suicide rates in 46 US states and Washington, DC, from 1981 to 2019.

## Introduction

Suicides are an increasing public health crisis in the US, and firearms are the most common method of death by suicide.^1,2^ In 2022, firearm suicides represented 54.6% of all suicides, with 27 032 firearm suicide deaths in the United States, amounting to around 74 deaths per day.^1^ Firearm suicide disproportionately impacts communities in poverty.^3,4,5^ Poverty, low-income, and unemployment are associated with greater exposure to chronic stress, thus leading to increased risk of poor mental health and suicide.^6,7,8,9,10^ Policies that target poverty alleviation and promote employment have the potential to address underlying and upstream determinants of firearm suicide (eg, poor mental health, chronic stress).

In the US, the federal earned income tax credit (EITC) is the largest poverty alleviation program for working families with low income. The EITC allows those with low income to pay less or no federal tax.^11^ The federal EITC is refundable, meaning that if the credit exceeds income tax liability, the International Revenue Service will refund the balance.^12^ The credit received by EITC beneficiaries as a tax refund is based on pretax earnings, marital status, and the number of children in the household.^12^ The EITC is received as an annual tax refund, and in the 2019 tax year, the average amount of EITC received was around $2461. Starting in 1986, states started introducing state-level credits on top of the federal credit, typically as a percentage of the federal rate (“EITC generosity”).^13^ Not all state EITCs are refundable, meaning individuals receive a tax break, yet no balance is refunded to the individuals. State EITCs were implemented in different years and vary in their generosity between states and over time, creating the opportunity to examine the association of state EITCs with health at the state population level. As of August 2024, 31 states plus Washington, DC, Guam, and Puerto Rico had enacted state EITCs ranging in generosity from 3% to 125% of the federal law (around $73.80 to $3076.25).^13^ EITCs have been associated with a decrease in poverty and an increase employment, along with overall improvement of mental and physical health.^14,15^

A few studies have found an association between refundable EITCs and suicide.^6,16,17^ One study^16^ found that a 10 percentage-point increase in state EITC was associated with a 4% reduction in suicide attempts and a 1% reduction in suicide deaths. Another study^6^ found that introducing generous state EITC is associated with a 3.9% decline in age-adjusted suicide rates. Lastly, a study^17^ found that EITCs are associated with a reduction in nondrug related suicides, particularly among women and those with a low-income. These studies also found that EITC generosity is important, and that states with a higher EITC generosity had a greater reduction in suicide.^6,16,17^ One study^18^ did not find an association between EITCs and suicide.

While studies have examined the association between EITCs and suicide,^6,16,17,18^ there is no mention of the association specifically with firearm suicide in the context of suicide and suicide mechanisms. The main pathways underlying the association between EITC laws and suicide are employment and poverty, and poverty is also associated with both firearm mortality and suicide.^6,19^ There is motivation to focus specifically on the determinants of firearm suicide because there are circumstances that differentiate firearm suicide and other means of suicide, including the availability and accessibility of firearms, along with the lethality of firearms.^20^ For example, because firearm use is more likely to lead to fatality than other suicide means, it is important to consider that a more generous EITC may have a more pronounced impact on firearm suicide compared with other suicide means in states where firearm ownership is high. Thus, when evaluating the association of state social policies (eg, EITC) with suicide, distinguishing firearm suicide from other means of suicide is useful. We sought to quantify the association of the generosity and presence of refundable EITC with firearm suicide from 1981 through 2019 using analytic approaches that accounted for staggered timing of EITC policy implementation.^21^ We hypothesized that state refundable EITC status is associated with a reduction in firearm suicide rates and an increase in the generosity of state refundable EITC is associated with a greater reduction in firearm suicide rates.

## Methods

### Study Design

We estimated the association between state refundable EITCs and firearm suicide rates from 1981 through 2019 using an ecological cohort design. Our study included 46 states and Washington, DC, covering 39 years over which 20 states implemented a refundable EITC (Table 1). In our study period, Vermont was the first state to implement a refundable EITC in 1988, with Maine having the most recent refundable EITC implementation in 2016 (Table 1).^22^ This study followed the Strengthening the Reporting of Observational Studies in Epidemiology (STROBE) reporting guideline. The institutional review board at the University of Washington did not require approval or informed consent for this study given the use of publicly available, deidentified data.

**Table 1.** Year of Implementation and Generosity of State Refundable Earned Income Tax Credits (EITC)^a^

State	Year of implementation^b^	EITC generosity (%)
Vermont	1988	23-32
Wisconsin^c^	1989	11-63
New York^d^	1994	7.5-30
Massachusetts	1997	10-23
Kansas	1998	10-18
Maryland^e^	1998	10-28
Indiana	1999	3.4-9
Washington DC^f^	2000	10-40
New Jersey	2000	10-37
Illinois	2003	5-18
Rhode Island	2003	5-15
Nebraska	2006	8-10
Oregon	2006	5-8
Iowa	2007	7-15
New Mexico	2007	8-10
Louisiana	2008	3.5
Michigan	2008	6-20
Connecticut	2011	23-30
California^g^	2015	85
Maine	2016	5

^a^ Data obtained from the Tax Policy center.^42^

^b^ Year of refundable EITC tax implementation.

^c^ Wisconsin’s credit is only available to workers with qualifying children.

^d^ New York City has an additional EITC that is 5% of the federal credit.

^e^ Maryland also offers a nonrefundable EITC set at 50% of the federal credit. Taxpayers may claim either the refundable credit or the nonrefundable credit. Maryland’s localities also offer an EITC.

^f^ The Washington DC EITC for childless workers is 100% of the federal credit and the range of eligible income is larger than the federal range.

^g^ California’s credit has a smaller range of eligible income than the federal credit at a maximum income at $30 000 for 2019.

### Exposure

The exposure was state refundable EITCs, measured by generosity and presence.^22^ EITC generosity was measured as the percentage of federal EITC and parameterized as a continuous variable, and coded as 0 for states without a refundable EITC. Refundable EITC status was parameterized as a binary variable with 1 equal to the presence of a state refundable EITC and 0 equal to the absence of a state refundable EITC. States were considered exposed at refundable EITC implementation and unexposed for states before refundable EITC implementation or a state that never enacted a refundable EITC. Consistent with the prior literature, states with nonrefundable EITCs were considered unexposed due to the lack of substantial rebate and limited association with health outcomes.^23,24,25,26^ We collected information on the presence and generosity of state EITC programs in all 50 states and Washington, DC, from 1981 to 2019. Four states (ie, Colorado, North Carolina, Minnesota, and Oklahoma) were excluded from the study because they had a refundable EITC that was revised to a nonrefundable EITC during the study period.

### Outcome

The outcome of interest was annual firearm suicide rates in each state. Individual-level data were not available. We collected annual, state-specific suicide rates using data from the US Centers for Disease Control and Prevention (CDC) Web-Based Injury Statistics Query and Reporting Systems, which are derived from the vital statistics death registry of the National Center for Health Statistics.^1^ The CDC does not report death rates when the absolute number of deaths in a state during a given year is fewer than 10, leading to 8 missing data points for the crude firearm suicide rate for Washington, DC, for the years 2000, 2004, 2009, 2011, 2013, 2016, 2017, and 2018 in our analysis. In a sensitivity analysis, we excluded Washington, DC, from the analysis (eTable 2 in Supplement 1).

### Statistical Analysis

We estimated the association between refundable EITC (measured by generosity and presence) and firearm suicide rates using heterogeneity-robust difference-in-difference (DID) and 2-way fixed effects (TWFE) regression specifications. As EITC laws were enacted in different years during the study period, the first regression used a heterogeneity-robust DID estimator.^21,27,28^ The second regression used TWFE estimation. We estimated an average exposure effect after EITC implementation. A taxpayer’s whole year of earnings are assessed before taxes are filed, and then EITC refunds are issued. Given the year lag in receiving EITC benefits, the indicators specifying exposure to EITC programs were lagged by 1 year in the analysis, as we considered the potential effect of a law only in the full first year after its implementation. All regression models used ordinary least squares and included state and year fixed effects and standard errors clustered at the state-level.

In the regression models, we adjusted for time-varying state-level covariates. We accounted for policy, economic, and demographic characteristics selected a priori based on the existing literature (eTable 1 in Supplement 1).^29,30,31,32,33,34,35^ We included a series of binary indicators equal to 1 if a state had implemented each of the following policies: Medicaid expansion under the Affordable Care Act, paid family leave, permit to purchase gun requirements, child access prevention laws, waiting periods, and minimum age requirements. The maximum Temporary Assistance for Needy Families benefits for a family of 3 was included as a continuous variable. We also adjusted for state-level economic characteristics (gross state product, state minimum wage) and state-level demographic characteristics (firearm ownership rates and percentage of population with high school diploma, living in a metropolitan statistical area, aged 15 to 24 years, married, a veteran, and adhering to a religion).

To assess the validity of the DID approach, we conducted an event study analysis. Event studies and the DID approach rely on the parallel-trends assumption. While this assumption cannot be verified, event study analyses can be used to assess potential violations of required assumptions by examining differences between exposed and unexposed states in years prior to implementation of a law. Another identifying assumption of our approach requires that there are no spill-over effects from exposed to unexposed states. We expect minimal spill over from state to state because both deaths and EITC refunds through state taxes are recorded based on state of residence; however, if an individual dies in 1 state but resided and filed taxes in another state, we would expect some spillover due to the way these data are recorded.

In recent years it has been noted that TWFE could be biased when there are multiple exposure periods and the effects vary over time.^36,37^ We implemented the Gardner heterogeneity-robust two-stage DID estimator using the did2s package in Stata version 17.0 (StataCorp).^21^ We have provided additional information on the Gardner heterogeneity-robust 2-stage DID estimator in the eMethods in Supplement 1). Data analysis was conducted from June 2022 through August 2024. Statistical significance was determined at the level of *P* < .05, and all tests were 2-sided.

## Results

### Firearm Suicide Over Time

Individual level data were not available in this study of state-level firearm suicide rates. In 2019, the final year of the study, the nationwide rate of firearm suicide was 7.29 per 100 000 persons. During the study period, the lowest rate of firearm suicide was 1.12 per 100 000 persons in Rhode Island in 2003, and the highest rate was 21.2 firearm suicides per 100 000 persons in Nevada in 1982.

### Event Study Findings

Adjusted event study specifications, which were used to evaluate the parallel trends assumption, are presented in the Figure. The Figure tested for differences pre- and postimplementation between states with refundable EITC and those without it. The event study detected minor and variable differences in firearm suicide rates many years before implementation (eg, 10 years prior to policy implementation: −0.24; 95% CI, −0.49 to −0.001), but did not detect differences in firearm suicide rates in the 5 years prior to policy implementation (eg, 5 years prior to policy implementation: −0.06; 95% CI, −0.26 to 0.13) (Figure). This suggests in the 5 years immediately preceding refundable EITC implementation, there were no detectable differences in the firearm suicide outcome in states that implemented refundable EITC compared with states that did not. An event study plot without covariate adjustment is presented in eFigure in Supplement 1.

**Figure.**
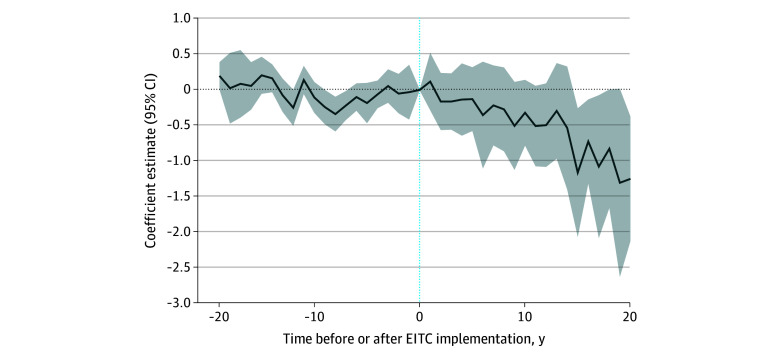
Adjusted Event-Study Plot of Earned Income Tax Credit (EITC) Presence^a^ ^a^The coefficient estimates with 95% CIs show the differences in outcomes between exposed and unexposed states over the years. This event study was based on heterogeneity-robust difference-in-difference regressions using Gardner 2-stage estimator, used ordinary least squares regression, included year and state fixed effects with standard errors clustered at the state level, and adjusted for time-varying state-level covariates. Presence of a refundable EITC was parameterized as a binary variable with 1 equal to the presence of a state refundable EITC.

### EITC and Firearm Suicide

We found a statistically significant negative association between refundable EITC generosity and firearm suicide rates (Table 2). When assessing the adjusted associations using heterogeneity-robust DID, compared to states without a refundable EITC, we found that every 10% increase in refundable EITC generosity was associated with a decrease in average firearm suicide rate by −0.28 (95% CI, −0.48 to −0.08) cases per 100 000 person-years, corresponding to a relative decline of 4.3% (95% CI, 1.5% to 7.0%). The average firearm suicide rate was −0.49 (95% CI, −0.91 to −0.07) cases per 100 000 person-years fewer comparing post-implementation and pre-implementation years among states that implemented a refundable EITC contrasting with the change during that time in states without a refundable EITC (Table 2), corresponding to a relative decline of 7.9% (95% CI, 2.7% to 12.9%). The TWFE regression models did not find an association between EITC and firearm suicide (Table 2).

**Table 2.** Difference-in-Difference (DID) Estimates for the Association of State Refundable Earned Income Tax Credit (EITC) Status and Generosity With Firearm Suicide Rates

Model description and exposure	DID estimate (95% CI)^a^	*P* value
**Regression 1: heterogeneity-robust DID^b^**		
EITC generosity^d^	−0.28 (−0.48 to −0.08)	.01
EITC presence^e^	−0.49 (−0.91 to −0.07)	.02
**Regression 2: 2-way fixed effects** ^c^		
EITC generosity	−0.14 (−0.42 to 0.14)	.31
EITC presence	−0.28 (−0.65 to 0.09)	.14

^a^ The DID estimate indicates the absolute change in firearm suicide rates per 100 000 per year. For example, every 10% increase in EITC generosity was associated with a decrease in the firearm suicide rate by 0.28 cases per 100 000 person-years.

^b^ Heterogeneity-robust difference-in-difference regressions used Gardner 2-stage estimator, used ordinary least squares regression, included year and state fixed effects with standard errors clustered at the state level, and adjusted for time-varying state-level covariates.

^c^ Two-way fixed effect difference-in-difference regressions used ordinary least squares regression, included year and state fixed effects with standard errors clustered at the state level, and adjusted for time-varying state-level covariates.

^d^ EITC generosity was measured as a percentage of the federal EITC and parameterized as a continuous variable.

^e^ Presence of a refundable EITC was parameterized as a binary variable with 1 equal to presence of a state refundable EITC and 0 equal to absence of a state refundable EITC.

## Discussion

This study provides new evidence about the association between EITC and firearm suicide. We found that each 10% increase in EITC generosity was associated with 0.28 fewer cases of firearm suicide per 100 000 people per year, corresponding to a relative decline of about 4.3%. To place these findings in context, the average amount of EITC received nationwide in 2019 was about $2461. The magnitude of these observed associations is in line with previous research. For example, 1 study found that 10% increase in EITC generosity was associated 0.23 to 0.24 fewer cases of suicide deaths per 100 000 people per year.^16^ Similarly to the present study, Morgan et al^16^ used an ecological approach to identify the association of state-level EITC with population-level rates of the outcome. An increase in generosity amounts to a larger tax credit, and potentially stronger association with poverty reduction and, thus, firearm suicide. Our study estimated these findings using a heterogeneity-robust DID approach, and the estimates did not hold when using a TWFE approach. These differences are likely because TWFE DID estimates may be biased under heterogeneous treatment effects when multiple treatment or exposure periods and exposure effects vary over time.^36,37,38^

This study adds to the body of evidence that social and economic policies may be an important contributor to reductions in suicide.^6,9,10,16,17,18,19^ One study assessed the association between state minimum wage laws and suicide and found that a $1 increase in state minimum wage was associated with a 1.9% decrease in the annual age-adjusted suicide rate.^10^ Similarly, another study found that generous unemployment insurance benefits reduce the impact of economic hardships, and unemployment benefits may be protective against suicide by providing a social safety net for all workers at risk of unemployment and their families, mitigating the negative mental health effects of job insecurity.^9^ While previous research indicates that poverty-alleviation programs reduce suicide rates, our study shows that they could do so specifically for firearm suicide.

Poverty is associated with increased rates of firearm-related deaths, including firearm suicide.^3,4,39^ One study investigated the association between state expansion of the Supplemental Nutrition Assistance Program (SNAP) and firearm suicide and found that state expansion of SNAP eligibility was associated with a decrease in the rate of firearm suicides.^19^ State expansion of SNAP eligibility may contribute to reductions in poverty and food insecurity, both of which are key risk factors for poor mental health and suicide.^19^ Our results further add to the evidence for the association of poverty-alleviating programs and firearm deaths by demonstrating that state-refundable EITCs (which also contribute to poverty reduction) are associated with reductions in firearm suicide rates and have the potential to address upstream determinants of risk factors associated with firearm suicide. There are various mechanisms in which EITC could address upstream determinants of firearm suicide. For example, there is conflicting evidence of EITCs’ association with mental health and depressive symptoms.^7,8,40^ It is possible that the EITC dollar amounts may not be large enough to be associated with improvements in mental health in some circumstances or populations and that EITC refunds may be associated with firearm suicide via other mechanisms. Future work should explore the mechanisms between poverty-alleviating programs and firearm suicide to accurately inform policies and interventions to mitigate the implication of poverty for firearm-related deaths.

### Limitations

This study has limitations. First, the results do not allow for causal inferences regarding the potential association of EITC participation with firearm suicide at the individual level. Second, even though we are using modern DID methods, there may still be outstanding methodological limitations when using state-level panel data, including estimates that do not include the true average effect size or are underpowered.^41^ Third, the study population included all individuals and was not limited to only those receiving EITCs, and these results may underestimate the association of EITC among those who are eligible for receiving EITCs. However, we still found that refundable EITCs are significantly associated with reductions in firearm suicide rates. Fourth, while we controlled for many time-varying, state-level factors that could have changed over the study period differentially by state and that are plausibly associated with firearm suicide, residual confounding may still be present in our findings.

## Conclusions

In this cohort study of state EITCs and firearm suicide, we found that an increase in refundable EITC generosity was associated with a decrease in firearm suicide rates, supporting the growing body of literature that highlights the association of antipoverty policies, such as state EITCs, with reducing firearm suicide rates. The association between EITCs and firearm suicide is likely complex, and multiple social and economic policies may be needed to reduce the rising rates of firearm suicide in the US. Policy makers interested in improving the health and well-being of communities and reducing the social and economic burden of firearm suicide should consider the implementation of more generous EITC programs. Findings may also be of interest to health care clinicians who are increasingly committed to improving health while addressing its social determinants.

## Notes

Supplement 1. **eTable 1. **Covariates and Data Sources **eTable 2. **Difference-in-Difference Estimates For the Association of State Refundable EITC Status and Generosity With Firearm Suicide Rates With Removing Washington DC **eMethods. **Gardner’s 2-stage Difference-in-Difference Estimator **eFigure 1. **Crude Event-Study Plot of Earned Income Tax Credit Presence Using Heterogeneity-Robust Difference-in-Difference

Supplement 2.
Data Sharing Statement
