# Supplementary material for: State Earned Income Tax Credits and Firearm Suicides
Source: JAMA Netw Open. 2025 Mar 21;8(3):e251398. doi: 10.1001/jamanetworkopen.2025.1398 (PMC11929027; doi:10.1001/jamanetworkopen.2025.1398)
Supplement: Supplement 1. — eTable 1. Covariates and Data Sources eTable 2. Difference-in-Difference Estimates For the Association of State Refundable EITC Status and Generosity With Firearm Suicide Rates With Removing Washington DC eMethods. Gardner’s 2-stage Difference-in-Difference Estimator eFigure 1. Crude Event-Study Plot of Earned Income Tax Credit Presence Using Heterogeneity-Robust Difference-in-Difference [file jamanetwopen-e251398-s001.pdf]

Supplemental Online Content

Asa N, Ellyson A, Rowhani-Rahbar A. State earned income tax credits and firearm suicides. *JAMA Netw Open*. 2025;8(3):e251398. doi:10.1001/jamanetworkopen.2025.1398

eTable 1. Covariates and Data Sources
eTable 2. Difference-in-Difference Estimates For the Association of State Refundable EITC Status and Generosity With Firearm Suicide Rates With Removing Washington DC
eMethods. Gardner's 2-stage Difference-in-Difference Estimator
eFigure 1. Crude Event-Study Plot of Earned Income Tax Credit Presence Using Heterogeneity-Robust Difference-in-Difference

This supplemental material has been provided by the authors to give readers additional information about their work.

© 2025 Asa N et al. *JAMA Network Open*.

eTable 1. Covariates and Data Sources

**Covariate**	**Data Source**
Maximum Temporary Assistance for Needy Families Benefits of three	University of Kentucky, Center for Poverty Research[1]
State minimum wage (or the federal minimum wage if it exceeded the state's minimum wage)	University of Kentucky, Center for Poverty Research
Gross state product	University of Kentucky, Center for Poverty Research
State expansion of Medicaid under the Affordable Care Act	Kaiser Family Foundation[2]
Paid family leave	National Partnership for Women and Families[3]
Percentage population with a high school degree	Kids Count Data Center[4]
Percent of population that are veterans	American Community Survey[5]
Percentage of the population living in a metropolitan statistical area	American Community Survey
Percent of population 15 to 24 years of age	American Community Survey
Percent of population married	American Community Survey
Firearm ownership	RAND[6]
Permit to purchase gun laws	RAND
Child access prevention laws	RAND
Waiting period laws	RAND
Minimum age restriction laws	RAND
Percent of population that adheres to a religion	Association of Religious Data Archives[7]

1. University of Kentucky Center for Poverty Research. Accessed June 4, 2022. http://ukcpr.org/resources/national-welfare-data
2. Medicaid. KFF. Accessed June 4, 2022. https://www.kff.org/medicaid/
3. National Partnership for Women and Families. Accessed June 4, 2022. https://nationalpartnership.org/wp-content/uploads/2023/02/state-paid-family-leave-laws.pdf
4. Kids Count Data Center. Annie E. Casey Foundation. Accessed June 4, 2022. https://datacenter.kidscount.org/
5. American Community Survey Data. Accessed June 4, 2022. https://www.census.gov/programs-surveys/acs/data.html
6. RAND. State Firearm Law Navigator. Accessed April 11, 2022. https://www.rand.org/pubs/tools/TLA243-2-v2.html
7. Quality Data on Religion. The Association of Religion Data Archives. Accessed June 4, 2022. https://www.thearda.com/

eTable 2. Difference-in-Difference Estimates For the Association of State Refundable EITC Status and

© 2025 Asa N et al. *JAMA Network Open*.

Generosity With Firearm Suicide Rates With Removing Washington DC

Model Description	Exposure	DID estimate[a]	p-value
**Regression 1**			
Heterogeneity-robust DID[c]	EITC generosity[e]	-0.24 (95% CI, -0.45 to -0.03)	0.03
	EITC presence[f]	-0.42 (95% CI, -0.85 to -0.01)	0.046
**Regression 2**			
Two-way fixed effects[d]	EITC generosity	-0.05 (95% CI, -0.28 to 0.17)	0.64
	EITC presence	-0.21 (95% CI, -0.57 to 0.16)	0.25

[a]The DID estimate indicates the absolute change in firearm suicide rates per 100,000 per year. For example: for every 10% increase in EITC generosity, the firearm suicide rate decreased by 0.24 cases per 100,000 person-years.

[b] Heterogeneity-robust difference-in-difference regressions was based on Gardner's two-stage estimator, used ordinary least squares regression, included year and state fixed effects with standard errors clustered at the state level, and adjusted for time-varying state-level covariates

[c] Two-way fixed effect difference-in-difference regressions used ordinary least squares regression, included year and state fixed effects with standard errors clustered at the state level, and adjusted for time-varying state-level covariates

[d] EITC generosity was measured as a percentage of the federal EITC and parameterized as a continuous variable

[e] Presence of a refundable EITC was parameterized as a binary variable with 1= presence of a state refundable EITC and 0= absence of a state refundable EITC.

© 2025 Asa N et al. *JAMA Network Open*.

eMethods. Gardner's 2-stage Difference-in-Difference Estimator

We used Gardner's two-stage heterogeneity-robust difference-in-difference estimator. The two-stage approach is robust to treatment-effect heterogeneity when adoption of the treatment is staggered. The first stage consists of a regression of outcomes on group and period fixed effects using untreated observations. The second stage estimates the group and period fixed effects which are then subtracted from observed outcomes. These adjusted outcomes are then regressed on treatment status. Under the parallel trends assumption, this procedure identifies the overall treatment effect, even when average treatment effects are heterogeneous over groups and periods. Additional information can be found in the following paper: Gardner J, Butts K, Wang T. Two-stage differences in differences. Published online July 13, 2022. https://arxiv.org/abs/2207.05943v1

© 2025 Asa N et al. *JAMA Network Open*.

eFigure 1. Crude Event-Study Plot of Earned Income Tax Credit Presence Using Heterogeneity-Robust Difference-in-Difference[a,b,c]

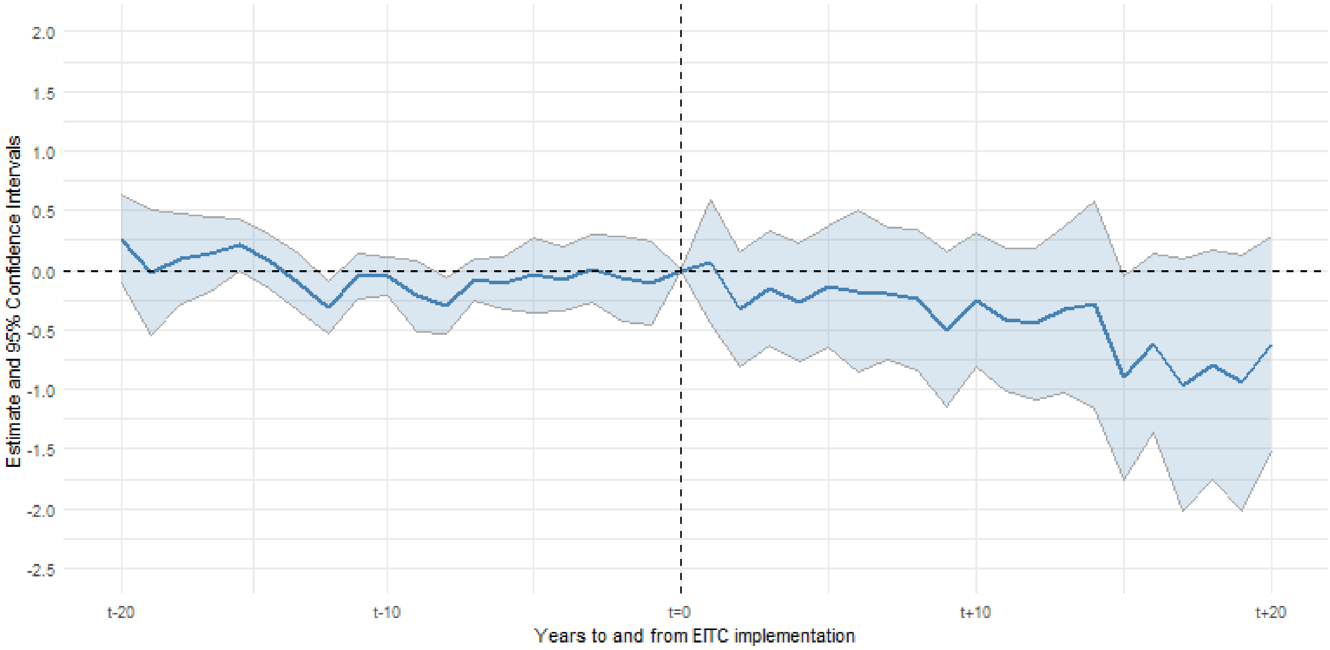

[a] The coefficient estimates with 95% confidence intervals show the differences in outcomes between exposed and unexposed states over the years. t= year of implementation.
[b] This event study was based on heterogeneity-robust difference-in-difference regressions using Gardner's two-stage estimator, used ordinary least squares regression, included year and state fixed effects with standard errors clustered at the state level
[c]Presence of a refundable EITC was parameterized as a binary variable with 1= presence of a state refundable EITC

© 2025 Asa N et al. *JAMA Network Open*.
