# Supplementary material for: State Earned Income Tax Credits and Firearm Suicides
Source: JAMA Netw Open. 2025 Mar 21;8(3):e251398. doi: 10.1001/jamanetworkopen.2025.1398 (PMC11929027; doi:10.1001/jamanetworkopen.2025.1398)
Supplement: Supplement 2. — Data Sharing Statement [file jamanetwopen-e251398-s002.pdf]

Data Sharing Statement

Asa. State Earned Income Tax Credits and Firearm Suicides. *JAMA Netw Open*. Published March 21, 2025. doi:10.1001/jamanetworkopen.2025.1398

Data
Data available: No
